# Higher craving and pleasure in response to exercise-related cues in patients with anorexia nervosa and exercise dependence

**DOI:** 10.1192/j.eurpsy.2026.12031

**Published:** 2026-07-31

**Authors:** M. Di Mauro, M. De Stefano, R. Ceres, R. Pecoraro, G. Cascino, C. La Femina

**Affiliations:** Università degli Studi di Salerno, Salerno, Italy

## Abstract

**Introduction:**

Physical activity is common in patients with anorexia nervosa (AN), who often reported symptoms consistent with exercise dependence. Craving, the subjective urge to engage in a specific behavior, is an important mechanism in the development of addiction and could be triggered by cues from the environment.

**Objectives:**

Aim of the present study was to investigate craving in people with AN with respect to the presence of exercise dependence in response to video cues of physical activity and sedentary behaviors, comparing them to a sample of healthy women.

**Methods:**

A total of 57 women with AN and 20 healthy women completed the Exercise Dependence Scale – Revised (EDS-R). According to EDS-R, 21 women with AN were classified as non-dependent asymptomatic (NDA), 21 as non-dependent symptomatic and 15 as dependent. All healthy women were NDA. All participants were exposed to video cues depicting subjects engaging in exercise and sedentary activities, and filled in the Crave Experience Questionnaire at three timepoints: at baseline, after exposure to sedentary activity videos, and after exposure to exercise activity videos.

**Results:**

Patients with AN displayed higher craving after exposure to physical exercise videos. Dependent participants reported higher intensity and intrusiveness of craving after exposure to exercise activity videos, compared to all other groups . Finally, we found an association between pleasure evoked by physical activity cues and the craving triggered by these videotapes (b = 0.1, p = 0.01).

**Conclusions:**

Our study suggests that craving is an existing dimension of exercise dependence in AN, supporting the positive reinforcement provided by exercise. This may indicate the importance to assess physical exercise dependence in people with AN and the potential efficacy of therapies that aim to reduce craving in the management of problematic physical activity in AN.

**Disclosure of Interest:**

None Declared

